# Is Sentinel Lymph Node Biopsy Feasible in Multicentric Breast Cancer? A Case Report and Literature Review

**DOI:** 10.3390/life15071018

**Published:** 2025-06-26

**Authors:** Mihaela Camelia Tîrnovanu, Elena Cojocaru, Vlad Gabriel Tîrnovanu, Elena Țarcă, Loredana Toma, Bogdan Florin Toma, Sorana Anton, Ștefan Dragoș Tîrnovanu, Roxana Ana Covali, Cipriana Ștefănescu, Irena Cristina Grierosu

**Affiliations:** 1 Department of Mother and Child Medicine-Obstetrics and Gynecology, Faculty of Medicine, “Grigore T. Popa” University of Medicine and Pharmacy, 700115 Iasi, Romania; mihaela.tirnovanu@umfiasi.ro (M.C.T.); sorana.anton@umfiasi.ro (S.A.); 2 Department of Morphofunctional Sciences I, Faculty of Medicine, “Grigore T. Popa” University of Medicine and Pharmacy, 700115 Iasi, Romania; elena2.cojocaru@umfiasi.ro (E.C.); bogdan.f.toma@umfiasi.ro (B.F.T.); 3 St. Josef Hospital, 65189 Wiesbaden, Germany; vlad.tirno@gmail.com; 4 Department of Surgery II, Faculty of Medicine, “Grigore T. Popa” University of Medicine and Pharmacy, 700115 Iasi, Romania; stefan-dragos.tirnovanu@d.umfiasi.ro; 5 Department of Medical Bioscience, Faculty of Bioengineering, “Grigore T. Popa” University of Medicine and Pharmacy, 700115 Iasi, Romania; loredana-toma@umfiasi.ro (L.T.); ana.covali@umfiasi.ro (R.A.C.); 6 Department of Morphofunctional Sciences II, Faculty of Medicine, “Grigore T. Popa” University of Medicine and Pharmacy, 700115 Iasi, Romania; cipriana.stefanescu@umfiasi.ro (C.Ș.); irena.raileanu@umfiasi.ro (I.C.G.)

**Keywords:** multilocular breast cancer, sentinel lymph node biopsy, axillary lymph node dissection, surgical treatment

## Abstract

Accurate lymph node staging is crucial for both prognosis (in the event of early-stage disease) and treatment (for local control of disease) in patients with breast cancer. Sentinel lymph node biopsy (SLNB) has been studied in numerous international trials, showing that it allows about 70% of axillary lymph node dissection (ALND) to be avoided and thus significantly reduces the morbidity associated with ALND. SLNB represents a necessary step in the diagnostic algorithm for breast neoplasms because the surgical treatment for breast cancer has become progressively less invasive. We present a case of a 70-year-old woman with multicentric breast cancer (MBC) treated by surgery at “Cuza Vodă” Women’s University Hospital, Iassy, Romania. In this case, only the ultrasonography established the diagnosis of left MBC with certainty. Conclusion: The detection of sentinel lymph nodes (SLNs) for MBC must be indicated. In this type of cancer, SLNB is accurate and practical, with sufficient quality control and interdisciplinary collaboration between surgical, nuclear medicine, and pathology units. Lymphoscintigraphy allows the patient to avoid axillary clearance surgery if the sentinel node is negative for metastatic disease. The variability of Ki67, PR, HER2, and ER status supports the idea that all individual foci should be tested in MBC cases to provide the best management and prognosis.

## 1. Introduction

Accurate lymph node staging is crucial for both prognosis (in the event of early-stage disease) and treatment (for local control of disease) in patients with breast cancer [1]. Axillary lymph node dissection (ALND) for breast cancer results in a high incidence of postoperative complications (the most important is arm lymphedema) that can reduce quality of life. Furthermore, in early breast cancer, almost 80% of axillary dissections expose no metastasis and, therefore, constitute useless surgery [2].

An important method in surgical oncology is, without a doubt, the sentinel lymph node (SLN) technique, which was first presented by Donald Morton and Alistair Cochran in 1992. Numerous international trials have assessed sentinel lymph node biopsy (SLNB), which has been shown to dramatically lower the morbidity associated with ALND by allowing for the avoidance of roughly 70% of ALND [3,4,5].

The localized nodes known as the SLN are responsible for the direct drainage of lymph from the main tumor. When the primary breast cancer is in its early stages (I or II), no imaging modality can accurately detect lymph node metastases. However, SLNB is a very dependable technique for screening axillary nodes and detecting metastatic disease, including micro-metastatic illness, in regional lymph nodes. The indications of the method, according to international guidelines, are as follows [6]: (1) breast neoplasm stage for invasive T1/T2 with clinically negative axilla; (2) in situ ductal carcinoma sufficient to require mastectomy or with suspected/proven micro-invasion; (3) patients with clinically negative axillary nodes following neoadjuvant chemotherapy. Contraindications to the method and implicitly to performing the sentinel lymph node biopsy refer to (a) clinically evidenced metastatic lymph node invasion; (b) breast neoplasm stage T3/T4 [7]; (c) inflammatory breast cancer; (d) previous major breast and axillary surgery or mammary and/or axillary radiotherapy because of significant alterations in lymphatic drainage [8]; (e) in situ ductal carcinoma when breast-conserving surgery is planned [9].

Multicentric breast cancer (MBC) has long been considered as a contraindication for SLNB due to concerns with sensitivity and greater false-negative rates [10]. Other reasons for false-negative results for SLN (missing or faint lymph node uptake) include tumor replacement of nodes, low doses of radiopharmaceuticals, poor quality radiopharmaceuticals, insufficient radiocolloid particles, too early or too late imaging times, and advanced patient age. As seen by guidelines from AGO, ASCO, and others, attitudes and recommendations regarding sentinel node biopsy in multifocal/multicentric breast cancer have changed dramatically over the last 14 years, and they currently support the procedure’s safety [9].

MBC is defined as two or more separate foci of breast carcinoma that are more than 2 cm apart within the same quadrant; MBC is the presence of separate autonomous foci of carcinoma in different quadrants [11], or a distance bigger than 4 cm. In industrialized countries, the incidence of MBC has been stated to be between 9 and 75% depending on the diagnostic criteria applied, while in Eastern European or less developed countries, the incidence is higher and can affect the average life expectancy [12,13]. The definition, occurrence, biological characteristics, prognostic importance, and subsequent consequences for therapy—particularly surgical therapy—are among the contentious aspects [14,15].

## 2. Case Report

We present a case of a 70-year-old woman with multilocular breast cancer treated with surgery at “Cuza Vodă” Women’s University Hospital Iassy, Romania. Her menarche occurred at the age of 14 years and menopause was surgical at 47 years after total hysterectomy with bilateral salpingo-oophorectomy for uterine fibroma. She had two vaginal deliveries, the first at 20 years old, and she breastfed for only 6 months. We must also mention the 10 abortions in her obstetrical history. The patient had no family history of breast cancer. As comorbidities, the woman had hypertension and grade II obesity with a body mass index of 34.6.

She detected a lump on her left breast 3 months before being admitted to the hospital. She was sent by the general practitioner to mammography. The imaging investigations performed by the patient were mammography, ultrasound exam, and magnetic resonance imaging (MRI). First of all, the digital mammography 2D was carried out in two incidences and performed in the left breast at a depth of 71 mm in the upper internal quadrant with high-intensity opacity, irregular contour, spiculated margins, polylobate, and a size of 16/20 mm with suspicious characteristics of malignancy—BIRADS 5.

The MRI examination with contrast substance was performed in the upper superior quadrant at 10 o’clock for a nodular mass with an irregular contour, with early intense contrast uptake, and wash-out curve kinetics with malignant appearance—BIRADS 6. Also, at the limit of the two upper quadrants, at 12 o’clock an area of architectural distortion of 25/15/8 mm with heterogeneous early contrast uptake with suspicious appearance for malignancy was found. The absence of suspicious adenopathy in the MRI for the left axilla was mentioned.

The core biopsy from the tumor localized in the upper internal superior quadrant specified the diagnosis of invasive breast carcinoma with no special type (NST) grading G3 (Nottingham score 8). The immunohistochemistry (IHC) found estrogen receptors (ERs), which were intensely positive in more than 90% of tumoral cells (Allred score 8); progesterone receptors (PR), which were intensely positive in approximately 30% of tumoral cells (Allred score 6); HER2neu (human epidermal growth factor receptor 2) 2+; and the proliferation marker Ki67, in 40% of the tumor cells. Because HER2 was equivocal (2+) in tumor A, unfortunately, reflex testing (FISH/SISH) was not performed at the external institution, despite our request for this.

**Figure 1:**
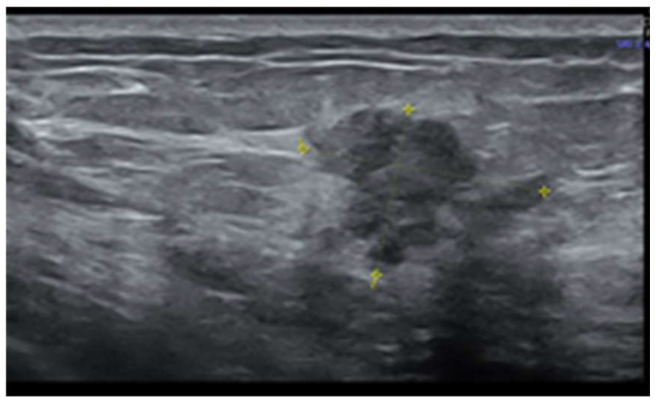
The hypoechoic inhomogeneous lump of 19/19/13 mm from the upper internal with irregular borders—tumor A.

**Figure 2:**
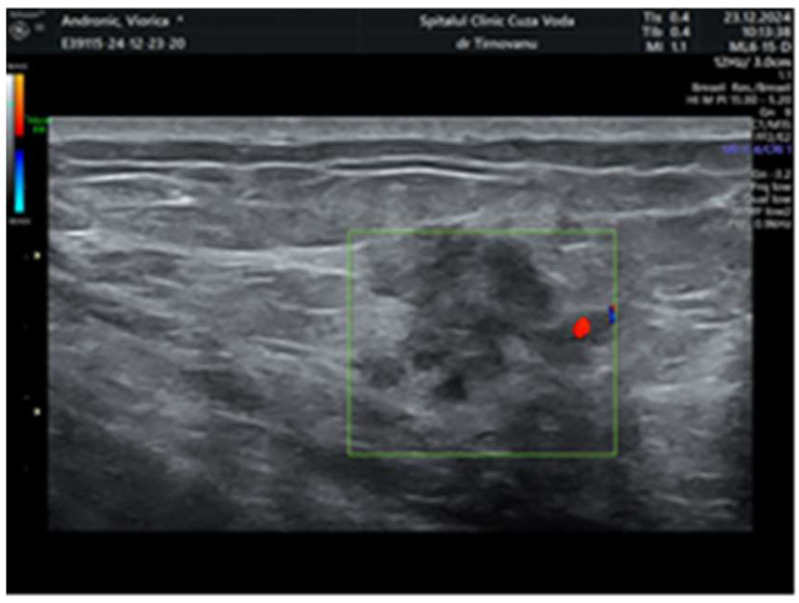
Vascular signal present at the periphery of the tumor A.

**Figure 3:**
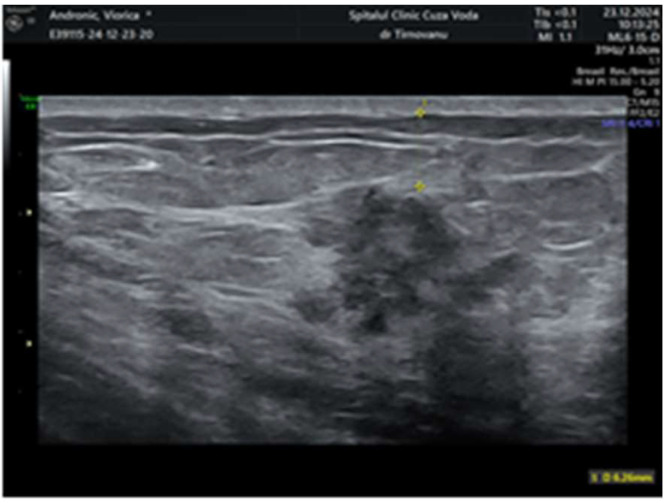
The distance between the bigger tumor and the skin.

**Figure 4:**
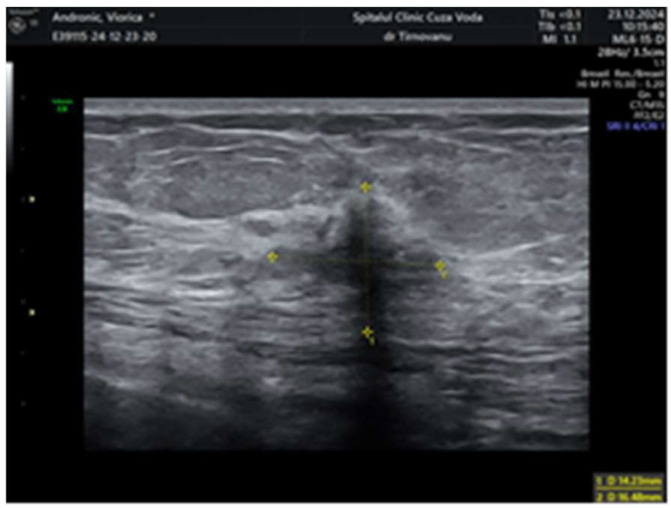
The dimensions of the second lump of 16/14 mm with heterogenous hypoechoic appearance—tumor B.

**Figure 5:**
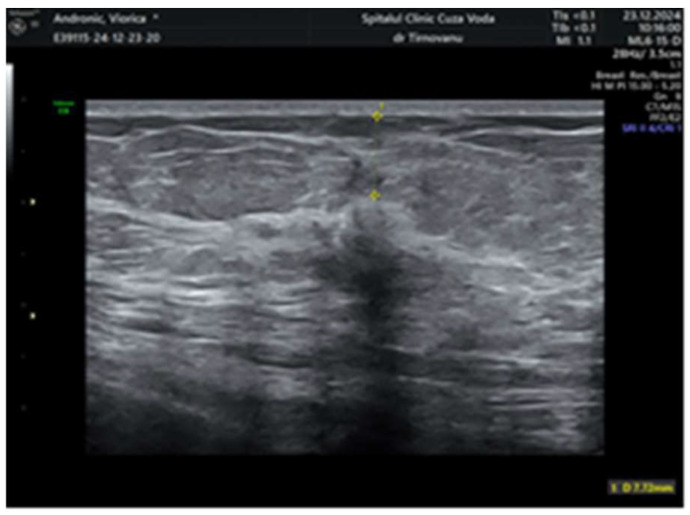
The distance between the second tumor and the skin.

Later, after a month, a second ultrasonography established the diagnosis of left multicentric breast cancer. It revealed in the left upper internal quadrant at 10 o’clock, at 5 cm from the nipple, a hypoechoic lump of 19/19/13 mm (anteroposterior/transversal/craniocaudal), with a horizontal axis bigger than the vertical one (Figure 1), with an irregular shape, inhomogeneous, with micro-lobulated margins, horizontal axis, vascular signal present (Figure 2), and posterior acoustic shadowing, and the distance of the tumor from the skin was 6.26 mm (Figure 3). We will name it tumor A. At the limit of the two upper quadrants, at 7 cm from the nipple, there was a second tumor of 16/14 mm (transversal/craniocaudal) with a heterogenous hypoechoic appearance (Figure 4, with a hyperechoic halo, without a vascular signal, and the distance of the tumor from the skin was 7.72 mm (Figure 5). We will name it tumor B. This second tumor corresponds in location to the area of architectural distortion described on the MRI. Therefore, both breast tumors did not comply with the ultrasound rule for malignancy, of “taller (rather) than broader”. The distance between breast tumors was 40 mm. The conclusion of the ultrasound exam was multicentric lesions in the upper quadrants of the breast, with characteristics of malignancy—BIRADS 5.

**Figure 6:**
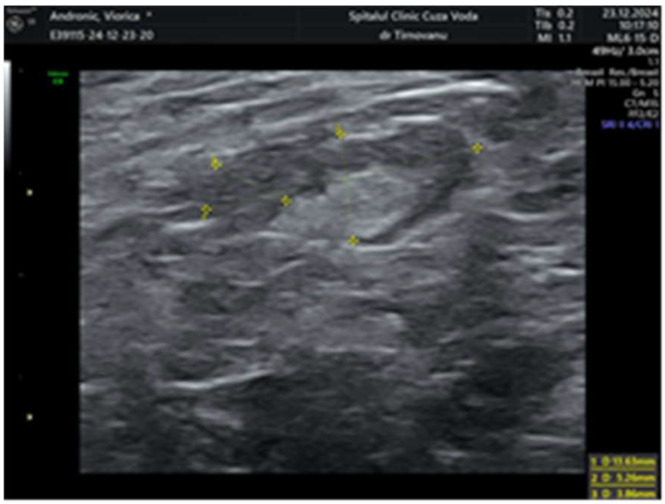
The axillary adenopathy of 13.6/5.2 mm with a preserved hilum and an increased cortex of 3.86 mm.

We reported the presence of lymph node enlargement at the base of the left axilla of 13.6/5.2 mm with a preserved hilum and an increased cortex of 3.86 mm (Figure 6). The preservation of the hilum is a benign sign, but an increased cortex by over 3 mm can be a malignant sign.

**Figure 7:**
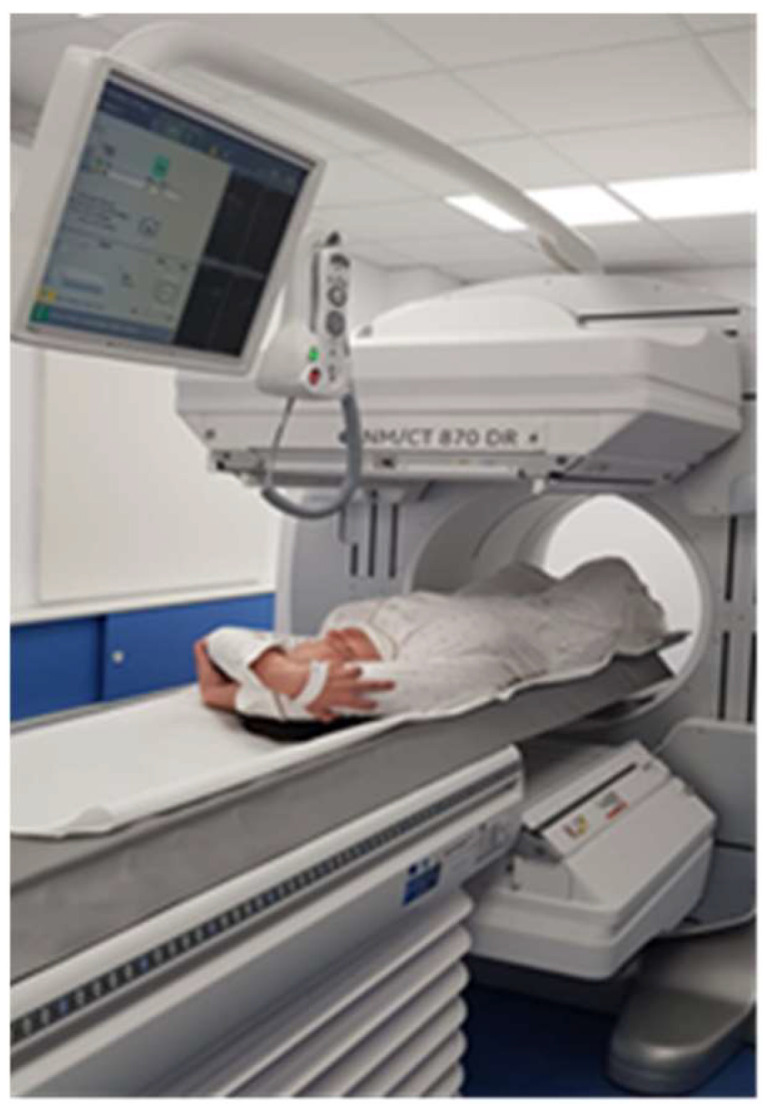
General Electric OPTIMA NM/CT 870DR SPECT-CT gamma camera which precisely locates (due to X-ray low-dose computed tomographic images) the functional abnormalities visualized in scintigraphy. Nuclear Medicine Laboratory, “Sfântul Spiridon” Clinic County Emergency Hospital, Iași.

**Figure 8:**
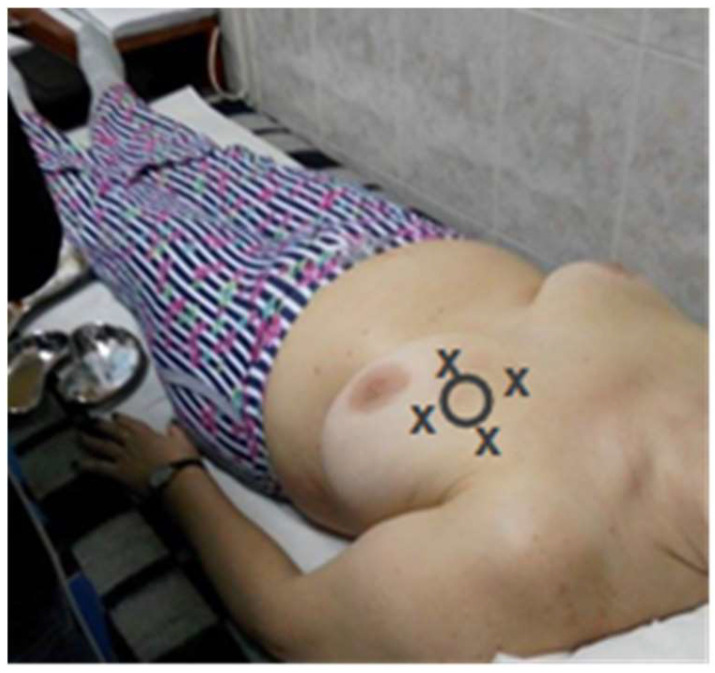
Schematic representation of the SLN technique: breast neoplasm (circle) and the peritumoral injection site of the radiopharmaceutical (marked with x).

**Figure 9:**
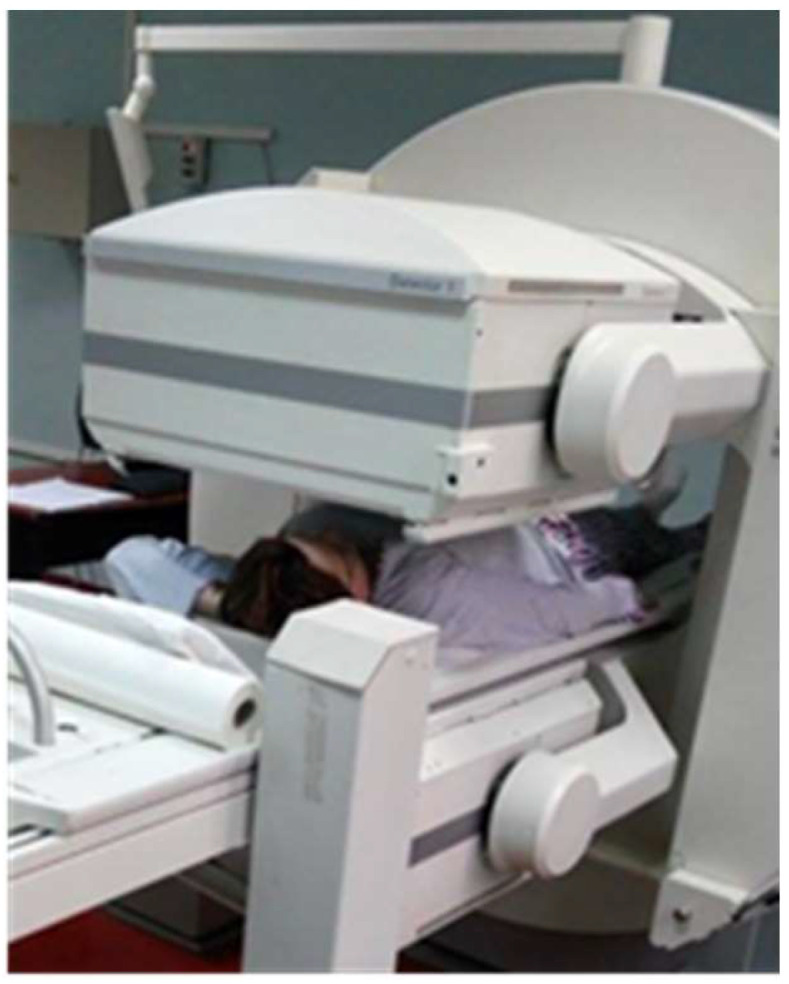
Acquisition of the lymphoscintigraphic image using a classical gamma camera dual detector, Siemens E.CAM from “Sfântul Spiridon” Clinic County Emergency Hospital.

The clinical exam upon admission to our hospital found a hard tumor of 3 cm located in the upper internal quadrant, which was imprecisely defined, painless, and mobile on deep and superficial tissues. Another smaller tumor of 1–2 cm was palpated at 12 o’clock on the limit between the upper quadrant, at 4 cm from the areola with the same clinical characteristics. No left axillary adenopathy was palpated. The patient was referred to the Nuclear Medicine Laboratory from the Clinic County Emergency Hospital to identify the SLN, using a General Electric Discovery SPECT-CT (single-photon emission-computed tomography-computed tomography) gamma camera (Figure 7, Figure 8 and Figure 9).

**Figure 10:**
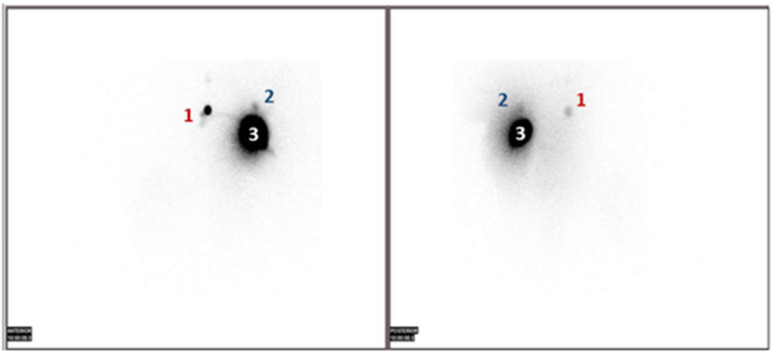
Detection by lymphoscintigraphy of the two SLNs—planar anterior and posterior view (SLN in the left internal mammary chain—1 and left axillary node—2); 3 marks the injected sites.

**Figure 11:**
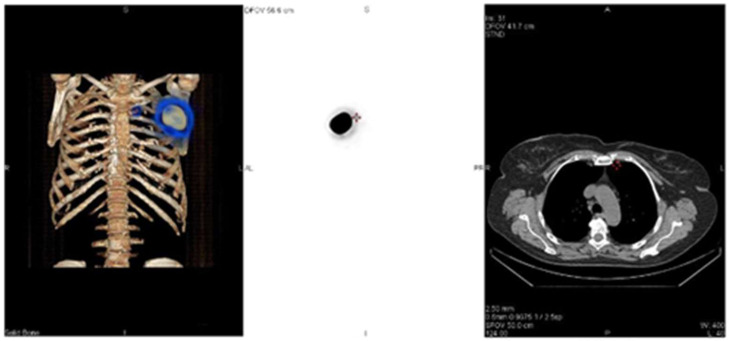
Detection by SPECT-CT of one of the two SLNs located behind the left chondrocostal joint II (bone reconstruction, nuclear medicine, and CT images); the intense area of fixation corresponds to the peritumorally injected radioactivity.

**Figure 12:**
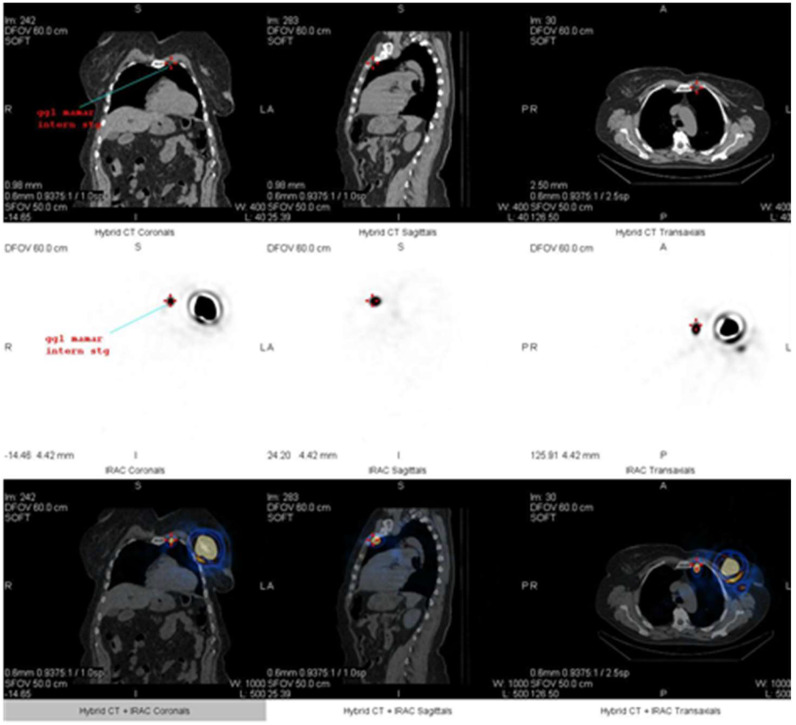
View of SPECT-CT with low dose of the SLN at the level of the left internal mammary chain: coronal, sagittal, and transverse images (CT, nuclear medicine, and fused series of images).

**Figure 13:**
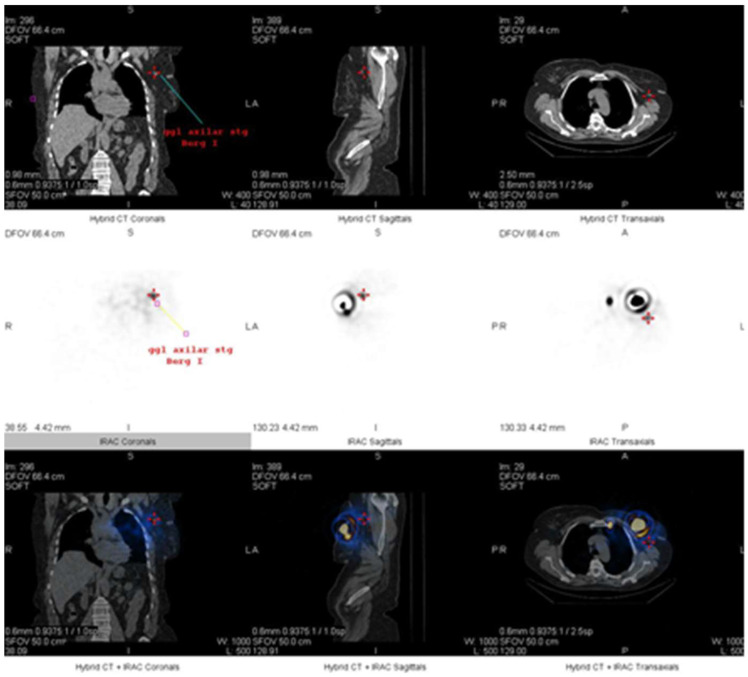
View at SPECT-CT of the SLN in the left axillary region: coronal, sagittal, and transverse images (CT, nuclear medicine, and fused series of images).

The gamma camera was armed with a low-energy, high-resolution collimator and peaked at 140 KeV, with a 20% window centered over the peak. Shielding of the injection site is typically not needed. ZOOM 1 and 256 × 256 matrix are often used. During the lymphoscintigraphy and SPECT-CT, first of all, two SLNs were identified; one behind the left chondrocostal joint II at the level of the left internal mammary chain (Figure 10, Figure 11 and Figure 12), and the other at the base of the axillary region (Figure 10 and Figure 13).

The patient was evaluated by the multidisciplinary team (MDT), which recommended upfront surgery, with no neoadjuvant therapy. The Ki67 of 40% for tumor A was noted, but the overall tumor characteristics and patient age led the team to favor surgical management.

**Figure 14:**
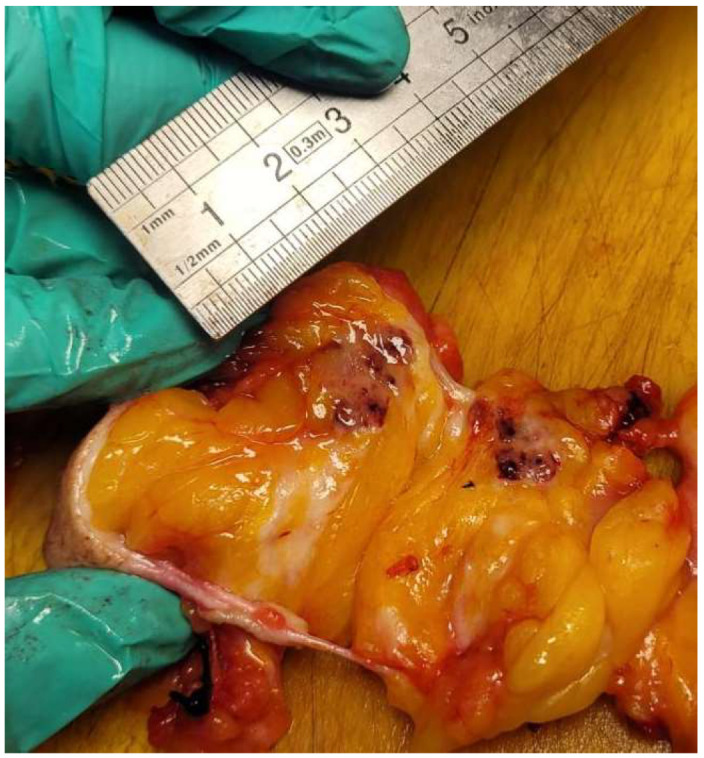
The macroscopical appearance of the first breast tumor (A) of 20 mm on the section.

**Figure 15:**
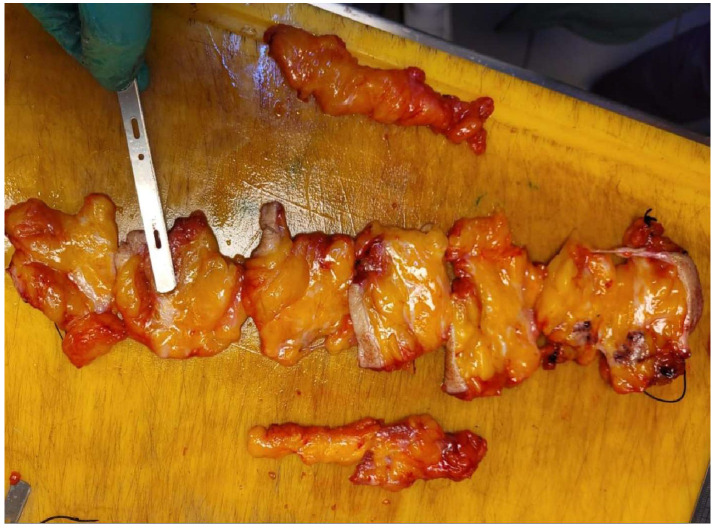
The second tumor (B) of 17 mm was detected after serial sections of the excised breast fragment, and shown by the pathologist.

The surgery was performed on the same day with a radiotracer injection after four hours. In the surgery room, we realized a partial mastectomy with the excision of both tumors. The biggest tumor, of 20 mm in concordance with the ultrasound dimension (Figure 14), was confirmed as invasive cancer by core biopsy preoperatively, but the small tumor was sent for a frozen section. The dimension of this second tumor was 17 mm, also in concordance with the ultrasound dimension (Figure 15), and was confirmed as malignant. The distance between the tumors was 45 mm, so the cancer was multicentric.

The second lesion was visualized as an architectural distortion on the MRI and later confirmed by ultrasound and surgery. A core biopsy was not performed initially due to imaging uncertainty, but the lesion was ultimately confirmed intraoperatively.

**Figure 16:**
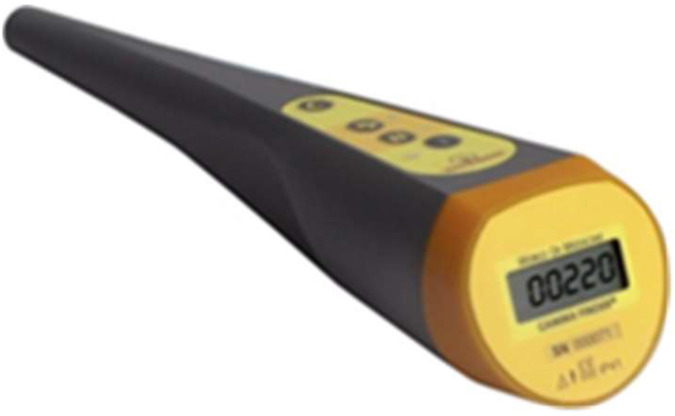
Gamma probe used in our clinic for intraoperative detection of the SLN.

**Figure 17:**
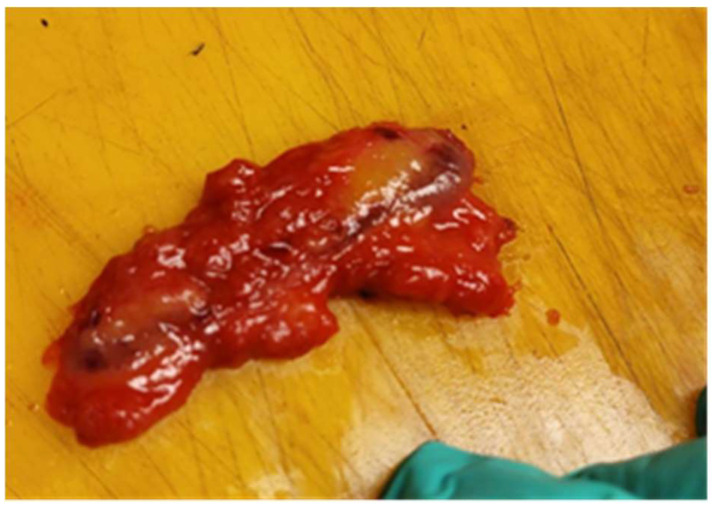
The macroscopical aspect of the axillary SLN in the section before the performance of the frozen section of half of it.

During surgery, we used a gamma probe with double detection, visual and acoustic, for identification of the SLN (Figure 16). The hottest area was identified by the pitch of the audible signal and the count rate on the digital display. At our patient, we identified the SLN from the left internal mammary chain after partial mastectomy. The intensity of the signal was 358 counters for this SLN. We realized an incision at the base of the left axillary zone of about 4 cm and found one SLN of approximately 2 cm with an intensity of the signal of 1500 counters. The frozen section for the biopsied SLN (Figure 17) specified that it be without metastases.

The internal mammary sentinel node was detected preoperatively via lymphoscintigraphy and SPECT-CT, but was not surgically excised due to its location, consistent with institutional limitations and common clinical practice. Only axillary sentinel nodes were excised.

We always check the intensity of the radioactivity of the removed SLN with the Gamma camera, by placing the probe on its surface to confirm the counts. The complete excision of SLNs was confirmed by counting the area after removing the purported sentinel node to see if the counts had fallen to background levels.

**Figure 18:**
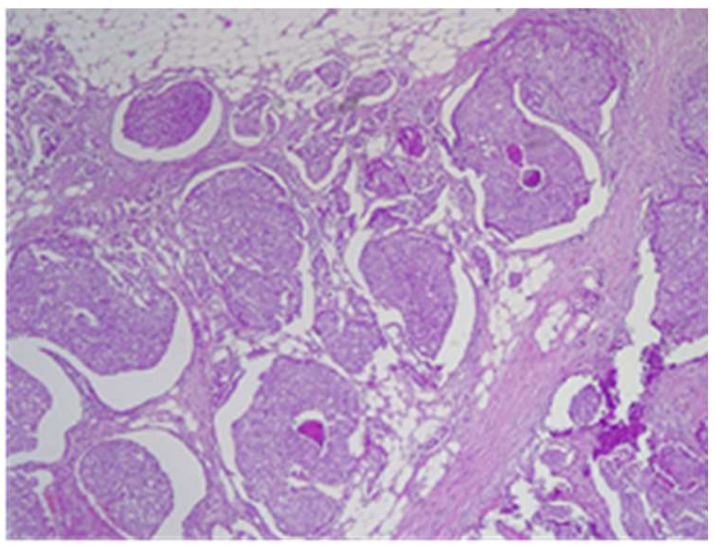
Tumor A—tumoral proliferation with solid architecture and central necrosis HE×4.

**Figure 19:**
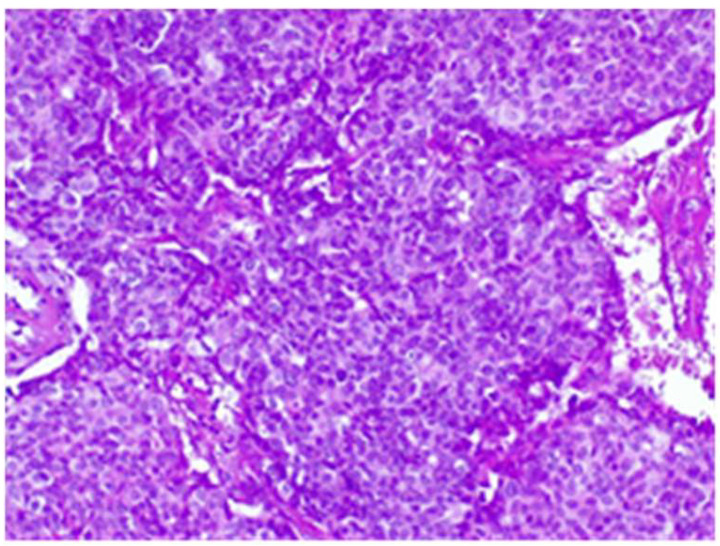
Tumor A with marked nuclear pleomorphism HE×20.

**Figure 20:**
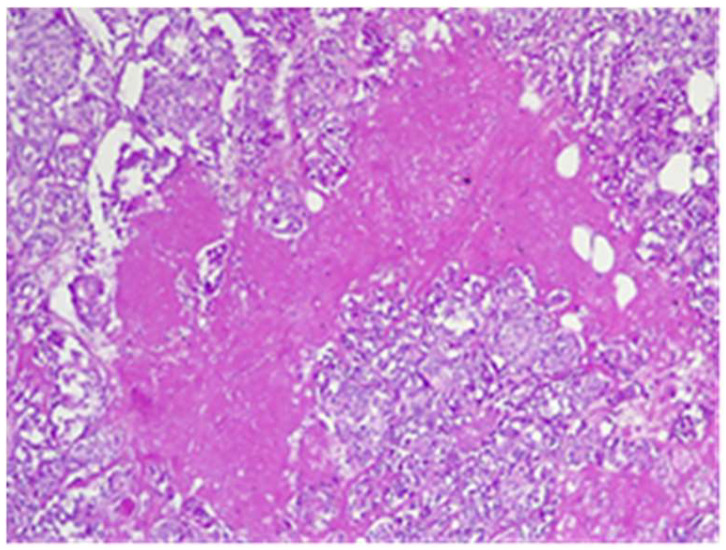
Tumor A—tumoral cells proliferation with central tumoral necrosis HE×10.

**Figure 21:**
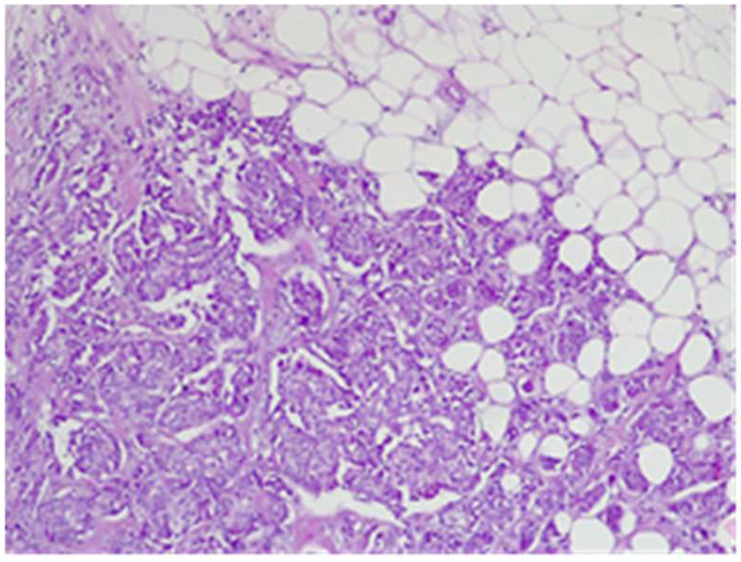
Tumor A tumor infiltration into adipose tissue HE×10.

The final histological result in paraffin established the malignancy of both tumors—ductal invasive breast cancer NST pT1c(m)N0G3L0V0pN1, but for tumor B, the pathologist requested IHC for e-cadherin to differentiate from a lobular cancer. Microscopically, tumor A at H&E (hematoxylin and eosin) showed tumor cells with solid (Figure 18), trabecular architecture, and focal in nests, as well as cords and cribriform aspect. The tumor cells show marked nuclear pleomorphism (nuclear score 3) (Figure 19) and high mitotic activity (approximately 13/10 hpf/field diameter 0.65). The tumor is associated with foci of tumor necrosis (Figure 20), reduced lymphocytic inflammatory reaction in the periphery of the tumor, and invasion in the adipose tissue (Figure 21), without clear aspects of angioinvasion.

**Figure 22:**
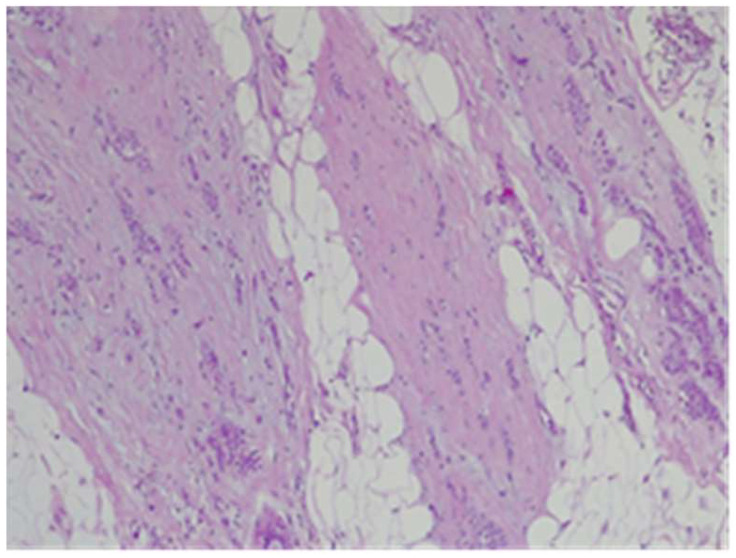
Tumor B cordonal proliferation with sclerohyaline stroma HE×10.

**Figure 23:**
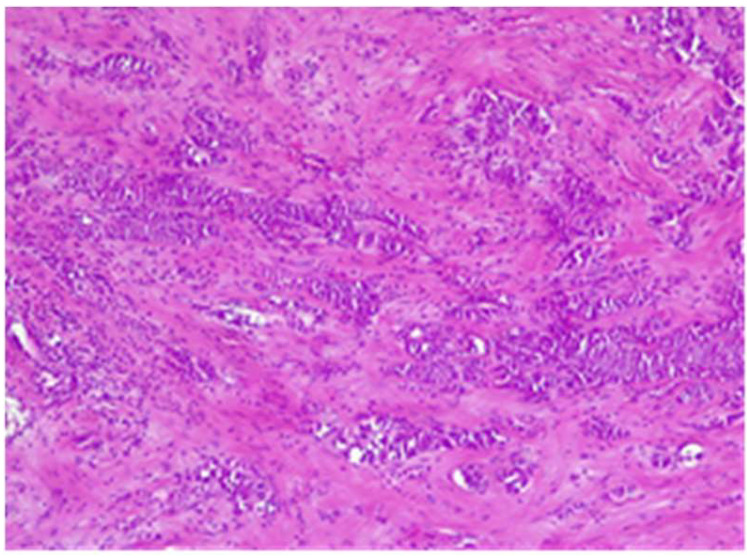
Tumor B trabecular proliferation with fibrous stroma HE×10.

**Figure 24:**
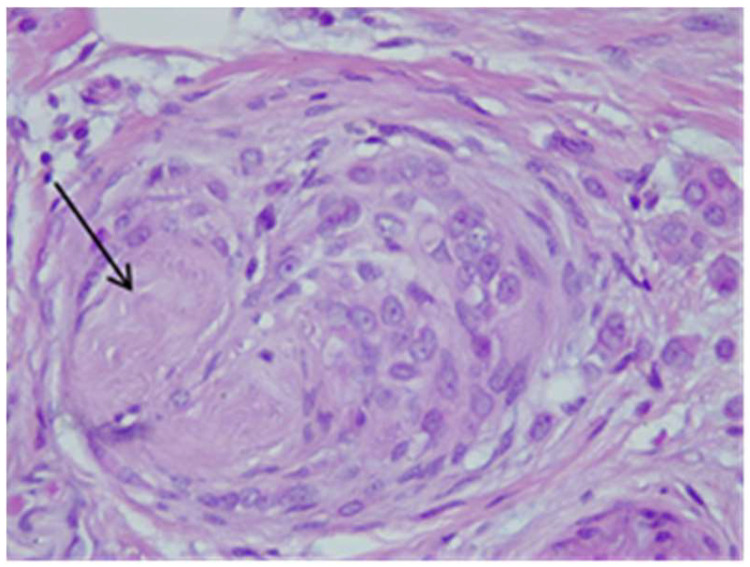
Tumor B—perineural invasion HE×40.

**Figure 25:**
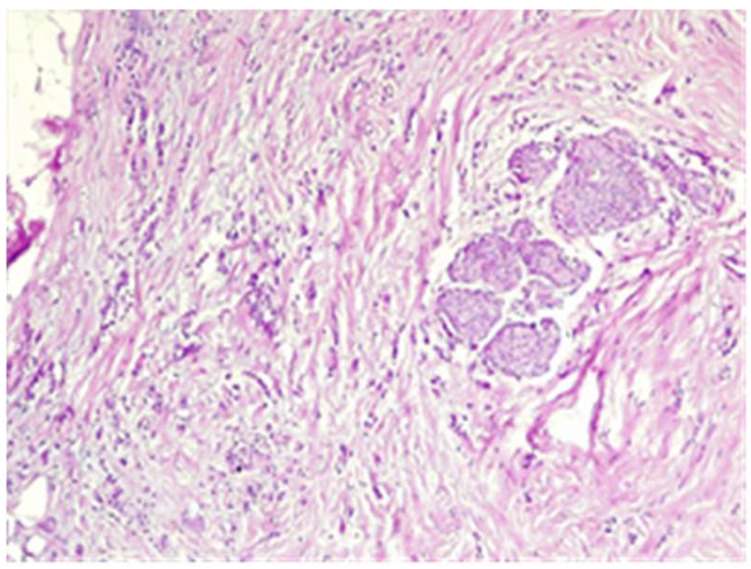
Tumor B—trabecular proliferation on the left, atypical lobular hyperplasia on the right HE×10.

Tumor B at H&E showed cells with cordonal (Figure 22) and trabecular (Figure 23) proliferation, in clusters or isolated, with moderate-to-marked nuclear pleomorphism (nuclear score 2) and reduced mitotic activity (1–2 mitosis/10 hpf-mitotic score 1). The tumor shows abundant fibrous stroma with reduced lymphocytic inflammatory infiltrate, images of intraductal carcinoma, with adipose and perineural tissue invasion (Figure 24), without clear aspects of angioinvasion. A focus of atypical lobular hyperplasia is visualized at the periphery (Figure 25).

**Figure 26:**
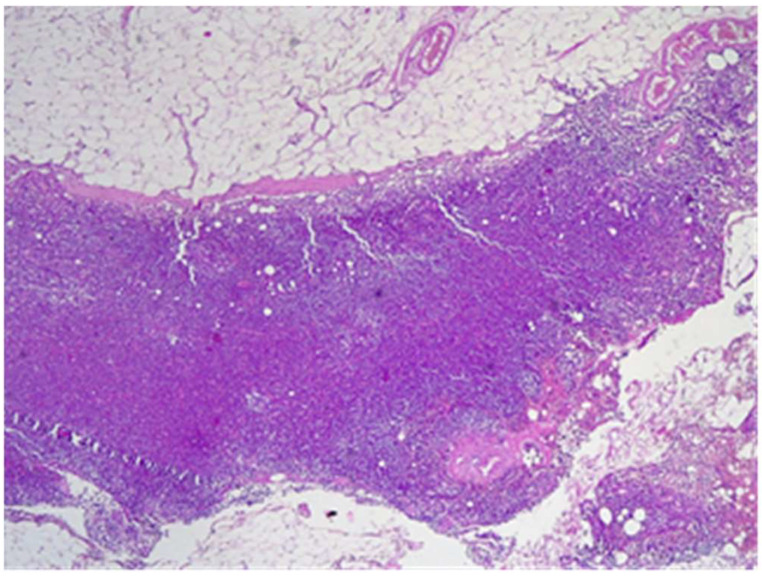
The sentinel lymph node without metastases HE×4.

The SLN was without metastases (Figure 26), with reactive changes and lipomatosis. In the neighboring adipose tissue, two more lymph nodes, 1–2 mm without tumor cells, were identified.

The IHC for tumor B established that was invasive lobular carcinoma type NOS, with e-cadherin negative in tumoral cells, so we had two different types of cancer in the same breast. Tumor B presents also p120-diffuse cytoplasmic labeling in tumor cells, Ck 5–6-positive, mosaic-type labeling. The carcinoma was intensely positive for ER (90% from tumor cells—Allred score 8), intensely positive for PR (90% from tumor cells—Allred score 8), and HER2-negative. The cell proliferation regulator Ki-67 was positive in approximately 10–15% of tumor cells. Regarding molecular profile, the expression was different in different tumoral foci, because Ki-67 had different values (Table 1). The invasive lobular carcinoma was type Luminal A and the invasive ductal carcinoma was Luminal B.

The patient underwent postoperative bone scintigraphy, which did not detect any bone changes. The osteotropic agent used for skeletal imaging is metastable technetium 99 (99mTc) labeled diphosphonates, and it is dedicated to first-level staging.

The patient received hormone therapy as the only adjuvant treatment, as decided by the oncologist based on her age and comorbidities. We understand that this may seem unexpected in a multicentric case, but the MDT decision was based on comprehensive clinical judgment.

## 3. Discussion

**Figure 27:**
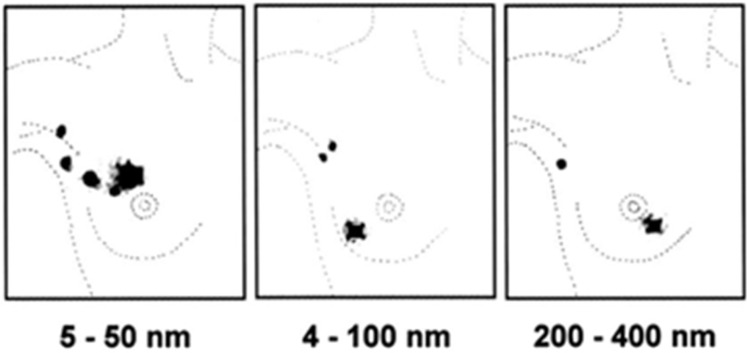
Migration of radiotracer from the injection site in breast tumors to lymph nodes as a function of radiocolloid sizes [17]. https://jnm.snmjournals.org/content/42/8/1198/F5 (accessed on 23 January 2025).

While some broad aspects of SLN procedures for breast cancer have been agreed upon, not all technical and practical details have been settled on. There are disagreements concerning the radiotracer’s particle size, the best injection route, the kind and time of scintigraphy, and intraoperative detection. An ideal tracer administration method for radio-guided SLNB is defined by three key factors: injection site, injected volume, and injected activity. The period between injection and operation is a fourth parameter that should be considered because it has a direct impact on the recommended dosage of radioactivity. Regarding the dimensions of radiocolloids and migration of the radiotracer from the injection site in breast tumors, those of relatively small dimensions, <50 nm, allow for the rapid visualization of lymphatic vessels a few minutes post-injection, but also of a larger number of lymph nodes. In contrast, radiocolloids >300 nm in diameter are preferentially retained in the sentinel node, exhibiting slow migration over time [16]. We exemplify this in Figure 27 [17]. Most physicians argue that the best compromise between rapid lymphatic drainage and ideal retention in SLNs can be found in a radiocolloid with particles that range in size from 100 to 200 nm [9].

The newest commercially available radiopharmaceutical used for radio-guided SLNB is Tilmanocept (Lymphoseek), composed of a dextran backbone with multiple glucose and mannose residues attached to DTPA for 99mTc labeling. Rapid transit from the primary site to the SLN and selective accumulation in that node, with prolonged retention and limited pass-through to second-echelon nodes, are two potential benefits of 99mTc-Tilmanocept’s small molecular size (7.1 nm) and receptor-targeted mannose moieties [18]. These features lead to high SLN visualization and high detection rates in comparison with sulfur colloid tracers [19]. Our colleagues from the Nuclear Medicine Laboratory use 99mTc NanoScan as a radiotracer, which contains human albumin colloidal particles (at least 95% of particles have a diameter ≤ 80 nm). This was the radiotracer that was administered to our patient.

**Figure 28:**
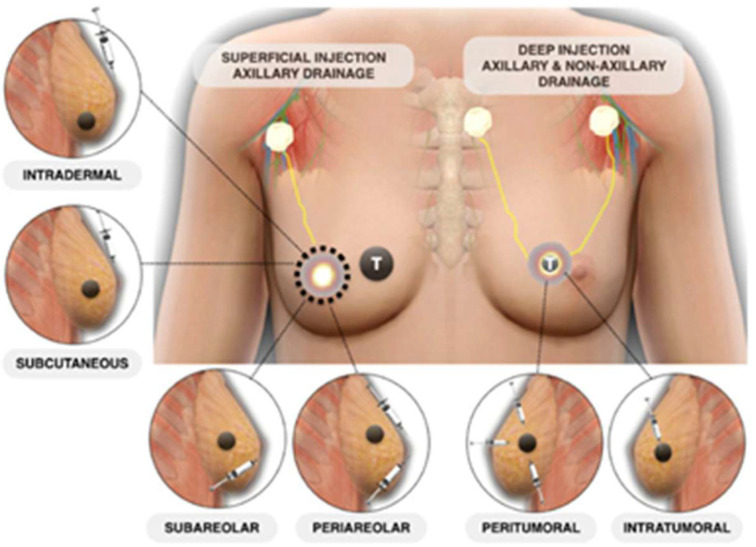
Radiotracer injection sites/modalities for SLN mapping and biopsy. On the left, superficial injections lead to almost exclusively axillary lymphatic drainage. On the right, deep tumor (T)-related injections lead to both axillary and nonaxillary lymphatic drainage [21].

Nowadays, depending on the amount of time between scintigraphy and surgery, a total injection of 5 to 30 MBq is usually regarded as enough for surgery scheduled for the same day [6]. According to what is now known, the ipsilateral axilla receives the majority of the multidirectional mammary lymphatic drainage, whereas the internal mammary chain lymph nodes achieve only 3% of the breast’s total lymph production [20]. Agreeing with this concept, we detected the location of the largest tumor in the upper internal quadrant in our patient, the SLN with a double location. Some authors, such as in [21], have recommended an intratumoral radiotracer injection for the detection of double lymphatic drainage (Figure 28). In our case, there was a double lymphatic drainage, and an internal mammary chain and ipsilateral axilla, revealed after peritumoral injection in four points.

Regarding the imaging procedures for the detection of SLN, preoperative lymphatic mapping has the potential to improve the accuracy of identifying SLN. Additionally, preoperative imaging acts as quality control for the use of the proper tracer, injection failure, radiopharmaceutical failure, and handling of the suitable breast and axilla. It has been demonstrated that SPECT-CT, when used in conjunction with planar imaging, can identify additional SLNs that are not visible on planar images in a significant proportion of patients, altering the drainage territory information in about 17% of cases [22]. The merged SPECT-CT scans give the surgeon valuable pre-operative supplementary information, such as improved localization, a shorter surgical time, and increased method confidence. For our patient, we used both methods of identification of the SLN and we had similar identification sites between planar and SPECT-CT images.

There are other methods for finding the SLN. Advanced tracers such as indocyanine green (ICG), superparamagnetic iron oxide (SPIO), and microbubbles have been discovered [23]. ICG does not involve the nuclear medicine department. Nonetheless, a navigation system for intraoperative fluorescence imaging and the ability to work in low light levels are required. Furthermore, ICG may leak in the surgical field following the resection of the first SLN, which results in the lymphatic vessels section, making it challenging to identify the subsequent SLN. Another noted drawback is the low molecular weight. Because ICG can spread more quickly than blue dye, SLN may needlessly be extensively dissected and removed [24]. The SPIO technique involves injecting a magnetic tracer, which is a contrast agent made of iron oxide crystal nanoparticles coated in carbohydrates. The tracer then migrates into the SLN through the lymph capillaries. The portable magnetometer that performs the detection creates an alternating magnetic field that momentarily magnetizes the SPIO and detects the magnetic response of the particles. After several months, the magnetic tracer is still observable despite gradually fading [25]. Because the magnetic tracer can be given as early as 15 days before surgery or right before the skin incision, this offers a convenient timeframe. The magnetic tracer’s brown hue can aid the surgeon in dissecting during surgery. The interpretation of postoperative breast magnetic resonance imaging may be complicated by void artifacts caused by the magnetic tracer’s intramammary persistence [26]. Patients who use pacemakers are also ineligible due to the magnetometer’s magnetic field, which might cause irregular heartbeats. Similarly, another technological limitation might be caused by metallic surgical equipment that interferes with ferromagnetic signals. Microbubble contrast-enhanced ultrasound imaging is an additional alternate method for SLNB. Following intradermal injection and lymphatic migration, phospholipid-stabilized microbubbles that contain sulfur hexafluoride gas function as sonographic contrast agents [27]. The contrast agent is inexpensive, and SLN can be visualized in real time. This method has a steeper learning curve, is operator-dependent, slower than the others, and necessitates skill in axillary ultrasound examination. It is less sensitive and has a lower detection rate than blue dye. Despite the concern about the risk of false-negative results for multicentric breast cancer, after examination with SLNB, axillary recurrence rates have been reported to be acceptable [10,28,29].

In a prospective multi-institutional trial from the Austrian Sentinel Node Study Group (ASNSG), SLNB evaluated the feasibility and accuracy of the detection of SN in 142 patients with multicentric cancer and compared this with data from 3216 patients with unicentric cancer. They found that SLBN is an indication for MBC without routine axillary lymph node dissection (ALND) [30]. Another study found that the 10-year overall survival for patients treated with sentinel lymph node dissection alone was not worse than the overall survival for patients treated with axillary lymph node dissection among women with T1 or T2 invasive primary breast cancer, no palpable axillary adenopathy, and one or two sentinel lymph nodes with metastases. Based on 10-year results, these results refute the usual use of axillary lymph node dissection in this patient population [31].

A study on 30 patients with MBC identified successfully the SLN in all 30 cases (identification rate 100%). Periareolar injection of radioisotope and blue dye was administered. Axillary metastases were present in 66.7% of cases (20/30). The sensitivity was 100% (20/20) and the false-negative rate was 0% (0/20). The negative predictive value was 100% (10/10), which is encouraging for patients [32]. In some hospital centers, the double labeling technique, a radiotracer, and a blue dye, is used, and the specificity of the technique has increased from 94 to 97% (radioisotopic labeling), to 99% (double labeling) [17]. A study detailed a long experience shows the role of radiocolloids in guiding SLNB as the technique of choice and standard-of-care procedure in patients with breast cancer meeting certain selection criteria [33,34,35,36,37]. In Romania, the use of blue dye is not approved by the national medicine agency, so we can use only the radiotracer technique.

According to a study by Freebody et al., multisite (triple-site) injection is associated with a high axillary sentinel lymph node identification rate. In the study, simultaneous injections of radiotracer at peritumoral, subcutaneous, and subareolar regions had an axillary sentinel lymph node identification rate of 98% in 123 breast lymphoscintigraphy patients [38]. For all cases of breast cancer, we performed lymphoscintigraphy (sentinel lymph node mapping) only by peritumoral injection with an SLN identification rate of 100%.

One study, which performed a meta-analysis of the literature evaluating the feasibility and accuracy of SLNB in multifocal and multicentric breast cancer in a total of 932 patients, established the accuracy rate, sensitivity, specificity, false-negative rate, and negative predictive value for SLN, which were 96.7%, 93.7%, 100%, 6.3%, and 93.5%, respectively. The authors concluded that, in a select subset of patients with multifocal and multicentric breast cancer, SLN biopsy can precisely predict lymph node status with false-negative rates comparable to that of women with unifocal breast cancer [39]. When a multifocal or multicentric breast cancer has an additional relative contraindication to performing SLNB, such as neoadjuvant chemotherapy or T > 5 cm, the false-negative rate increases [40]. Combining the visual evaluation of nodes with intraoperative gamma camera counting and digital palpation through the surgical incision is a good method of lowering false-positive and false-negative outcomes. The current staging system for MBC implies that each tumor that arises independently should be used to estimate the patient’s prognosis, based on the size of the largest deposit [41]. The Lang et al. study suggests that all foci of MBC should be considered to estimate the risk of recurrence and metastasis [42].

According to the current guidelines, ER/PR status should be assessed in the sample obtained at the initial core biopsy or the main tumor foci in multifocal breast cancer (more than one distinct tumor foci in a quadrant). We respected this rule for our cases. However, we assessed the receptor status after surgery of both individual foci in MBC and confirmed the variability.

E-cadherin is a crucial molecule for cell adhesion; numerous studies have shown that its down-regulation is linked to the advancement of breast cancer, worse prognoses, and treatment resistance [43,44,45,46]. Weissenbacher et al. stated that E-cadherin expression was significantly lower in MBC compared to the unifocal group and suggested the down-regulation of E-cadherin in MBC was causally connected with a worse prognosis [46,47,48]. In our case, E-cadherin was only analyzed for the lobular carcinoma foci and was negative.

The luminal B subtype is understood as the most aggressive form of ER+ breast cancer cases and often does not show benefits for hormone therapy [49].

Breast cancer is mainly diagnosed in early stages (90–95%); however, 20–30% of these patients become metastatic. Bone is the single most frequent site for bone metastasis in breast cancer patients [50]. Bone metastasis occurred within 3 years in 60.74% of cases after surgery, and after 3 years in 39.26% of cases [51]. On the other hand, 36% of the incidence of bone metastasis is from breast cancer [52], with a tendency of incidence in luminal subtypes (ER+/HER2− 58.52% and in ER+/HER2+ subtype 47.28% of incidence). With an incidence of bone metastases of 34.49% and 36.39%, respectively, the triple-negative (TN) subtype is more impacted by lung involvement (32.09%), whereas the ER−/HER2+ subtype has a larger percentage of liver metastases (31.72%) [53]. Molnar IA et al. show that the luminal A subtype presented a tendency of isolated bone metastases in 59% of cases [54]. Some studies suggest a higher likelihood of lobular breast cancer metastasizing to the bone [55,56].

Breast cancer women older than 65 years developed bone-only metastases 1.5 times more often than younger breast cancer patients did [56]. Marwa and Mubarak show that sixty percent of patients diagnosed with bone metastases were 45 years or younger [57]. Nevertheless, much is still unknown regarding bone metastasis formation. Based on the disruption of multicellular units (osteoblasts, osteoclasts, bone lining cells, and osteocytes) and the release of growth factors (TGF-B, FGF, PDGF, and IGF), the etiopathology of bone metastasis encourages the growth of tumor cells and compromises secondary bone architecture [52]. TGF fuels the further growth of breast cancer cells. Even if breast cancer cells preferentially activate osteoclasts, resulting in osteolytic metastasis, osteoblastic areas are, in general, also present [58]. Breast cancer cells also produce endothelin-1 (ET-1) that activates osteoblasts, resulting in the accumulation of immature mineralized bone (sometimes termed osteoid), and suppresses osteoclast activity [59]. Breast cancer can spread to any bone in the body, but the spine, hips, shoulders, skull, and upper parts of the leg bones near the hips are where it is most often found [60,61].

Reported sensitivity and specificity for bone scintigraphy are 78% and 48%, respectively [62]. Since bone turnover is typically detected by bone scintigraphy, metastases exhibiting a high prevalence of lytic activity may be regarded as a false-negative. An accumulation of agents on bone scans may result from a change in 5–10% of bone, which is not always connected to cancer. However, this can also be a confounding factor, with benign pathologies in such degenerative diseases. Because of this, borderline cases may necessitate a second-level exam. The lack of volumetric evaluation and low spatial resolution (less than 1 cm) are two other drawbacks of bone scans. Breast cancer patients should be monitored with bone scintigraphy, especially in the first 3 years after diagnosis, when they are at an increased risk of bone metastases; even more so if the molecular type of breast cancer is luminal subtype A or B.

## 4. Conclusions

In the presented case, only the ultrasonography established the diagnosis of left multicentric breast cancer with certainty. The detection of SLN for MBC must be indicated. Given suitable quality control and interdisciplinary cooperation of surgical, nuclear medicine, and pathology units, SLNB is feasible and accurate in this disease entity. Lymphoscintigraphy allows the patient to avoid axillary clearance surgery if the sentinel node is negative for metastatic disease. The conventional radioactivity-dependent technique of SLNB is enshrined in evidence-based algorithms for breast cancer management, a time-proven technique that has been refined and continues to improve by using SPECT-CT detection. SPECT/CT adds value, particularly in cases with negative, inconclusive, or equivocal results, and provides better and more accurate localization of sentinel nodes in patients presenting drainage outside of the ipsilateral axilla, like in our case. However, planar lymphoscintigraphy remains a satisfactory imaging option for imaging departments not equipped with SPECT/CT due to its good patient-based detection rate, a fact confirmed in our patient. The two tumors were histologically different; tumor A was invasive ductal carcinoma type NST, and tumor B was invasive lobular carcinoma type NOS. The variability of Ki67, PR, HER2, and ER status supports the idea that all individual foci should be tested in MBC cases to provide the best management and prognosis.

## Figures and Tables

**Table 1:** The molecular profile of both tumors.

Markers	Tumor A—Ductal Invasive Carcinoma	Tumor B—Lobular Invasive Carcinoma
ER	90% positive	90% positive
PR	30% positive	90% positive
HER2	2+	Negative
Ki67	40%	10–15%

## Data Availability

The data presented in this study are available on request from the corresponding author.
